# 放疗联合免疫治疗对肺癌脑转移的疗效和安全性的*meta*分析

**DOI:** 10.3779/j.issn.1009-3419.2022.101.48

**Published:** 2022-10-20

**Authors:** 利娟 徐, 应泰 陈, 梅 王

**Affiliations:** 1 215200 苏州，苏州市第九人民医院门诊部 Department of Outpatients, Suzhou Ninth People's Hospital, Suzhou 215200, China; 2 100076 北京，北京航天总医院胸外科 Department of Thoracic Surgery, Beijing Aerospace General Hospital, Beijing 100076, China; 3 100076 北京，北京航天总医院市场开发处 Department of Marketing, Beijing Aerospace General Hospital, Beijing 100076, China

**Keywords:** 放疗联合免疫, 疗效, 安全性, 脑转移, 肺肿瘤, *meta*分析, Radiotherapy combined with immunotherapy, Efficacy, Safety, Brain metastases, Lung neoplasms, *Meta*-analysis

## Abstract

**背景与目的:**

免疫治疗(immunotherapy, IT)被推荐用于治疗晚期非小细胞肺癌(non-small cell lung cancer, NSCLC)，而脑放疗(radiation therapy, RT)是脑转移(brain metastasis, BM)患者的主流治疗方法。本研究旨在调查RT和IT联合使用的疗效及安全性。

**方法:**

检索时限截至2022年5月1日，在中国知网、万方、PubMed、EMBASE、Cochranc数据库中进行了文献检索。异质性采用*I*^*2*^检验和*P*值进行判断。发表偏倚采用漏斗图评价。采用纽卡斯尔-渥太华量表(Newcastle Ottawa Scale, NOS)评估纳入研究的质量。采用Stata 16.0软件进行统计分析。

**结果:**

纳入17篇文献共涉及2,636例患者。在RT+IT组和RT组的比较中，总生存期(overall survival, OS)(HR=0.85, 95%CI: 0.52-1.38, *I^2^*=73.9%, *P*_异质性_=0.001)和颅内远距离控制(distant brain control, DBC)(HR=1.04, 95%CI: 0.55-1.05, *I^2^*=80.5%, *P*_异质性_ < 0.001)未发现明显差异，但RT+IT组颅内控制(local control, LC)优于RT组(HR=0.46, 95%CI: 0.22-0.94, *I^2^*=22.2%, *P*_异质性_=0.276)，发生放射性坏死/治疗相关影像学改变(radionecrosis/treatment related imaging changes, RN/TRIC)风险高于RT组(HR=1.72, 95%CI: 1.12-2.65, *I^2^*=40.2%, *P*_异质性_=0.153)。在RT+IT同步治疗组和序贯组的比较中，未发现OS(HR=0.62, 95%CI: 0.27-1.43, *I^2^*=74.7%, *P*_异质性_=0.003)和RN/TRIC(HR=1.72, 95%CI: 0.85-3.47, *I^2^*=0%, *P*_异质性_=0.388)在两组中存在差异。但同步治疗组DBC优于序贯治疗组(HR=0.77, 95%CI: 0.62-0.96, *I^2^*=80.5%, *P*_异质性_ < 0.001)。

**结论:**

RT联合IT并未改善NSCLC BM患者的OS，而且还会增加RN/TRIC的风险。此外，相对于RT与IT序贯治疗，RT与IT同步治疗可改善DBC的疗效。

## Abstract (en)

**Background and objective:**

Immunotherapy (IT) is recommended for the treatment of advanced non-small cell lung cancer (NSCLC), while brain radiotherapy (RT) is the mainstream treatment for patients with brain metastases (BM). This study aimed to investigate the efficacy and safety of combined use of RT and IT.

**Methods:**

The date was limited to May 1, 2022, and literature searches were carried out in CNKI, Wanfang, PubMed, EMBASE and Cochrane databases. Heterogeneity was judged using the *I*^*2*^ test and *P* value. Publication bias was assessed using a funnel plot. The quality of included studies was assessed using the Newcastle-Ottawa Scale (NOS). Statistical analysis was performed using Stata 16.0 software.

**Results:**

A total of 17 articles involving 2, 636 patients were included. In the comparison of RT+IT group and RT group, no significant difference was found in overall survival (OS) (HR=0.85, 95%CI: 0.52-1.38, *I*^*2*^=73.9%, *P*_heterogeneity_=0.001) and intracranial distance control (DBC) (HR=1.04, 95%CI: 0.55-1.05, *I*^*2*^=80.5%, *P*_heterogeneity_ < 0.001), but the intracranial control (LC) in the RT+IT group was better than the RT group (HR=0.46, 95%CI: 0.22-0.94, *I*^*2*^=22.2%, *P*_heterogeneity_=0.276), and the risk of radiation necrosis/treatment-related imaging changes (RN/TRIC) was higher than RT (HR=1.72, 95%CI: 1.12-2.65, *I*^*2*^=40.2%, *P*_heterogeneity_=0.153). In the comparison between the RT+IT concurrent group and the sequential group, no significant difference was found in OS (HR=0.62, 95%CI: 0.27-1.43, *I*^*2*^=74.7%, *P*_heterogeneity_=0.003) and RN/TRIC (HR=1.72, 95%CI: 0.85-3.47, *I*^*2*^=0%, *P*_heterogeneity_=0.388) was different between the two groups. However, DBC in the concurrent treatment group was better than that in the sequential treatment group (HR=0.77, 95%CI: 0.62-0.96, *I*^*2*^=80.5%, *P*_heterogeneity_ < 0.001).

**Conclusion:**

RT combined with IT does not improve the OS of NSCLC patients with BM, but also increases the risk of RN/TRIC. In addition, compared with sequential RT and IT, concurrent RT and IT improved the efficacy of DBC.

在我国，肺癌发病率和死亡率居各类恶性肿瘤首位^[1]^。其中，非小细胞肺癌(non-small cell lung cancer, NSCLC)占肺癌的80%以上。超过一半的肺癌患者首诊时被诊断为晚期或转移性肺癌^[2]^。脑同肺、肝和骨骼均为NSCLC的常见转移部位^[3]^，大部分NSCLC患者最终会发展为脑转移(brain metastases, BM)。BM是NSCLC常见且具有破坏性的并发症，极大程度降低了患者的生活质量，NSCLC BM患者的5年生存率约为2.9%^[4]^。目前针对NSCLC脑转移患者的主流疗法为局部治疗，如手术和放射治疗(radiotherapy, RT)，后者包括立体定向放射外科(stereotactic radiosurgery, SRS)、立体定向放射治疗(stereotactic radiotherapy, SBRT)和全脑放射治疗(whole brain radiotherapy, WBRT)，但效果并非十分理想。近年来应用于肺癌治疗的免疫药物逐步增多，程序性死亡受体-1 (programmed death protein-1, PD-1)和程序性死亡受体配体-1(programmed death-ligand 1, PD-L1)抑制剂也被推荐为晚期NSCLC一线免疫治疗药物^[5]^。免疫系统在RT的抗癌功效中发挥着关键作用，二者联合使用可以改善黑色素瘤患者BM预后，但在NSCLC BM的应用上仍处于探索阶段。目前，免疫疗法与RT联合使用对患者总生存期及安全获益程度仍存在争议。本研究旨在通过对RT+IT与单独RT比较的相关结局进行*meta*分析，以探讨两者之间的疗效及安全性。

## 1 资料与方法

### 1.1 检索策略

检索数据库中国知网、万方、PubMed、EMBASE、Cochrane等，检索时限为2022年5月1日以前发表的相关研究。英文检索词：“NSCLC”“brain metastasis”“radiotherapy”“immunotherapy”“immune checkpoint inhibitors”“PD-1”“PD-L1”采用主题词与自由词结合方法检索，中文检索词：“非小细胞肺癌”、“脑转移”、“放射疗法”和“免疫疗法”。同时，手工检索相关文献以确保纳入研究尽可能全面。语言限制为英文和中文。

### 1.2 纳入标准

① 研究对象为有1个或多个BM的NSCLC患者; ②所有患者均接受脑部放疗; ③至少一组患者使用PD-L1、PD-1或细胞毒T淋巴细胞相关抗原4(cytotoxic T lymphocyte-associated antigen-4, CTLA-4)免疫药物治疗; ④研究结果包括OS、颅内局部控制(intracranial local control, LC)或颅内远距离的比例大脑控制(intracranial distant brain control, DBC)和放射性坏死/治疗相关影像学改变(radiation necrosis/treatment related imaging change, RN/TRIC)。排除无法获取原始数据的文献、会议摘要和病例报告等。

### 1.3 提取数据

由两名研究者独立提取纳入研究的相关信息：第一作者、发表年份、国别、组别、患者人数、原位癌类型、脑放疗类型、免疫治疗药物、中位随访时间、各组别发生结局人数及总数或各结局风险效应值HR及95%CI等。效应值从纳入文献直接提取或通过*Kaplan-Meier*曲线计算^[6]^。若纳入文献同时计算了单因素和多因素HR值，则提取包含信息较多的多因素结果。

### 1.4 统计学方法

结果采用HR及95%CI进行评估。异质性采用*I*^*2*^检验和*P*值进行判断，当*P*≤0.1或*I*^*2*^≥50%时认为存在异质性，采用随机效应模型; 反之，采用固定效应模型。比较RT+IT组与单独RT治疗组的疗效和安全性，并根据RT与IT治疗时间间隔，将RT+IT组分为同步治疗组和序贯治疗组，同步治疗组时间间隔为1周-3个月。对于文献≥3篇的组别按照国别和是否调整做亚组分析。发表偏倚采用漏斗图评价，并进行敏感性分析评估结果稳定性。采用评价队列研究质量的纽卡斯尔-渥太华量表(Newcastle-Ottawa Scale, NOS)评估纳入研究的质量^[7]^。NOS表包含9个项目，满分9分，6分及以上认为质量较高可纳入分析。所有分析均采用Stata 16.0软件进行分析，除特殊说明外，双侧*P* < 0.05为差异有统计学意义。

## 2 结果

### 2.1 文献检索结果

通过中国知网、万方、PubMed、EMBASE、Cochrane共检索中英文文献1, 980篇，排除重复、不相关、会议摘要、数据不全及综述性文献后，最终共纳入17篇文献。具体检索过程见图 1。

**图 1:**
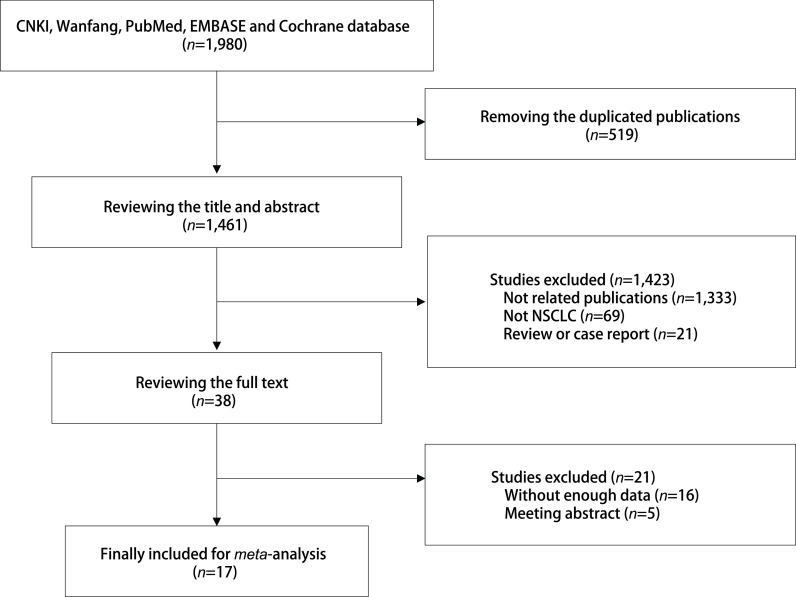
纳入文献筛选流程图 The studies searching flow chart

### 2.2 纳入文献基本特征

纳入17篇文献^[8-24]^共涉及2,636例患者。研究国别主要为美国。10篇文献研究OS结局，8篇文献研究RN/TRIC结局，6篇文献关于DBC结局，3篇文献关于LC结局。中位随访时间跨度为4.4个月-29.9个月。纳入文献NOS评分均高于6分，故纳入所有文献进行分析。见表 1。

**表 1:** 研究人群基本特征 General characteristics of the included studies

Author	Year	Country	Sample size	Median age (year)		Outcome	Cancer	Follow-up (mon)	Radiation	Median radiation dosage	Immunotherapeutic drugs	Treatment line	NOS
				Experiment	Control								
Colaco^[8]^	2016	U.S	180	-		RN/TRIC	NSCLC, others	11.7	SRS	-	PD-1, CTLA-4	NA	6
Chen^[9]^	2018	U.S	260	-		OS	NSCLC, others	9.2	SRS/SBRT	20 Gy/1 f	PD-1, CTLA-4	NA	8
Hubbeling^[10]^	2018	U.S	163	61	62	RN/TRIC	NSCLC	16	SRS/SBRT; PBI; WBRT	18 Gy/1 f; 30 Gy/10 f; 35 Gy/12 f	PD-1, PD-L1	2	9
Martin^[11]^	2018	U.S	480	61	62	RN/TRIC	NSCLC, others	23.1	SRS/SBRT; PBI; WBRT	18-20 Gy/1 f; 25-30 Gy/5 f	PD-1, CTLA-4	NA	8
Schapira^[12]^	2018	U.S	37	63		RN/TRIC	NSCLC	17.6	SRS/SBRT; PBI; WBRT	18 Gy/1 f	PD-1, PD-L1	NA	8
Doi^[13]^	2019	Japan	100	67		OS	NSCLC	4.4	WBRT	30 Gy/10 f	PD-1	NA	7
Koenig^[14]^	2019	U.S	97	68	63	OS, DBC, RN/TRIC	NSCLC, others	6.5	SRS/SBRT; PBI; WBRT	22 Gy/1 f	PD-1, CTLA-4, PD-L1	NA	8
Lanier^[15]^	2019	U.S	271	-		DBC	NSCLC, others	29.9	SRS	18 Gy/1 f	PD-1, CTLA-4, PD-L1	NA	6
Li^[16]^	2019	U.S	125	65		OS	NSCLC, others	> 6	SRS	-	-	2	7
Shepard^[17]^	2019	U.S	51	-		OS, DBC, RN/TRIC	NSCLC	7	SRS	-	PD-1, PD-L1	2	9
Enright^[18]^	2020	U.S	77	62	63	OS, LC, DBC	NSCLC	11.4	SRS/SBRT	30 Gy/5 f; 21-24 Gy/1 f	PD-1, PD-L1	NA	9
Guénolé^[19]^	2020	France	194	61	60	OS, LC, DBC	NSCLC, others	11.9	-	23.1 Gy/3 f; 33 Gy/3 f	PD-1, CTLA-4, PD-L1	NA	7
Lee^[20]^	2020	South Korea	51	62	59	OS	NSCLC	19.1	SRS	19 Gy/1 f	PD-1	NA	9
Singh ^[21]^	2020	U.S	85	61.9		OS, RN/TRIC	NSCLC	12	SRS/SBRT	18 Gy/1 f	PD-1, CTLA-4, PD-L1	2	7
Singh ^[22]^	2020	U.S	136	-		DBC	NSCLC	13.7	SRS	22 Gy/1 f	PD-1, PD-L1	NA	8
Kowalski^[23]^	2020	U.S	179	59	60	LC, RN/TRIC	NSCLC, others	9			PD-1, CTLA-4, PD-L1	2	7
Silvia^[24]^	2022	Italy	150	65	66	OS	NSCLC	21		18 Gy/1 f; 28.9 Gy/3 f	PD-1, PD-L1	NA	8
NOS: Newcastle Ottawa Scale; U.S: United States; RN/TRIC: radionecrosis/treatment related imaging changes; NSCLC: non-small cell lung cancer; SRS: stereotactic radiosurgery; SBRT: stereotactic radiotherapy; WBRT: whole brain radiotherapy; PD-1: programmed death protein-1; PD-L1: programmed death-ligand 1; OS: overall survival; DBC: distant brain control; CTLA-4: cytotoxic T lymphocyte associated antigen 4; NA: not available; LC: local control; PBI: partial brain irradiation.													

### 2.3 RT+IT组和RT组比较的*meta*分析结果

共7篇文献报道了两组与OS的关联，结果显示RT+IT治疗组的OS与RT治疗组无显著差异(HR=0.85, 95%CI: 0.52-1.38, *I^2^*=73.9%, *P*_异质性_=0.001); 5篇文献报道了两组与DBC的关联，未发现两组间DBC存在统计学差异(HR=1.04, 95%CI: 0.55-1.05, *I^2^*=80.5%, *P*_异质性_ < 0.001); 3篇文献报道了两组与LC的关联，结果显示，RT+IT治疗组LC优于RT治疗组(HR=0.46, 95%CI: 0.22-0.94, *I^2^*=22.2%, *P*_异质性_=0.276); 5篇文献报道了两组与RN/TRIC的关联，结果显示RT+IT组发生RN/TRIC风险高于RT组(HR=1.72, 95%CI: 1.12-2.65, *I^2^*=40.2%, *P*_异质性_=0.153)。详见图 2。

**图 2:**
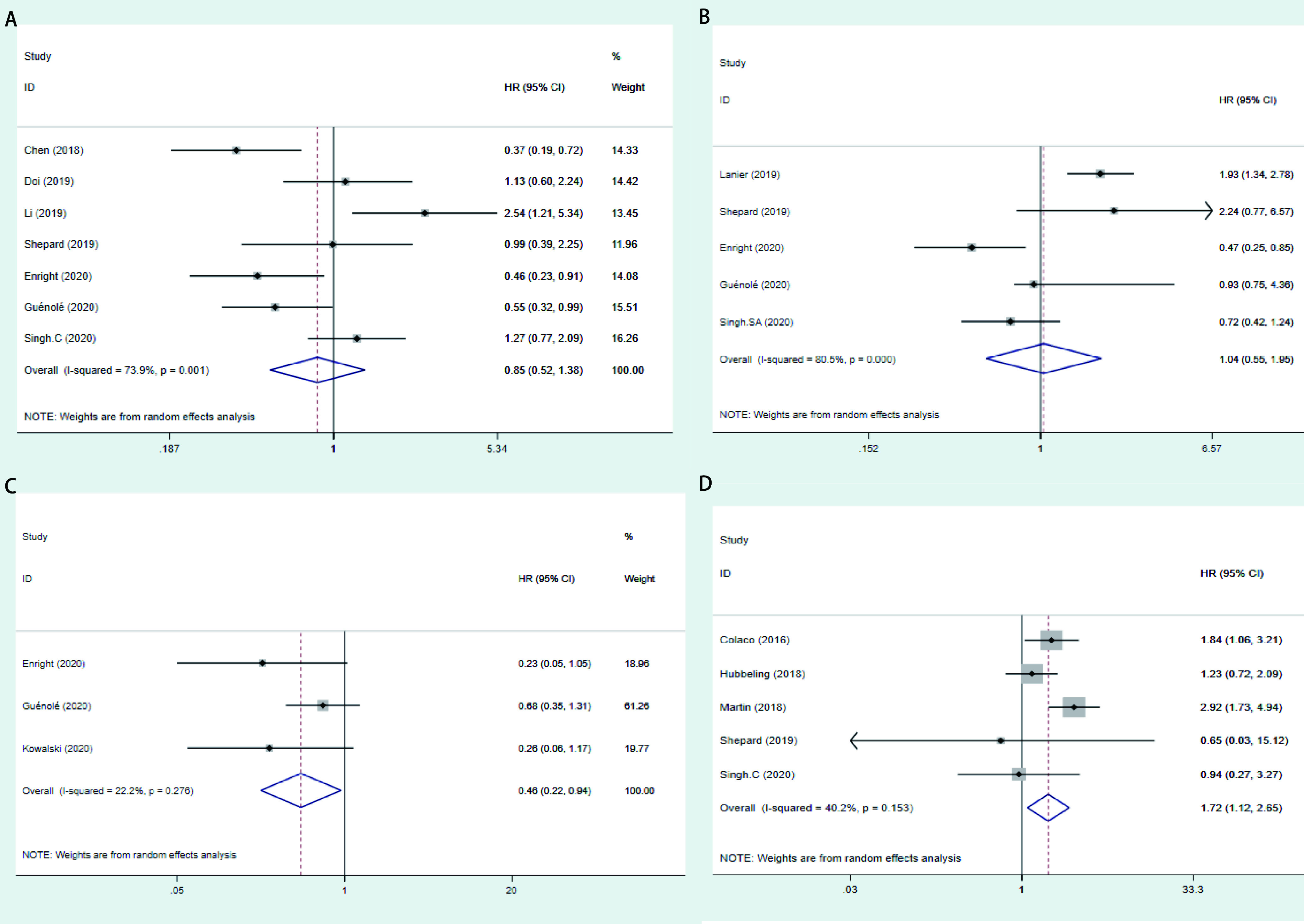
RT+IT组 *vs* RT组各结局的森林图。   A：OS; B：DBC; C：LC; D：RN/TIRC。 Forrest of RT+TT *vs* RT. A: OS; B: DBC; C: LC; D: RN/TIRC.

### 2.4 RT+IT同步治疗组和序贯组比较的*meta*分析结果

共5篇文献报道了两组与OS的关联，结果显示同步治疗组的OS与序贯治疗组无显著差异(HR=0.62, 95%CI: 0.27-1.43, *I^2^*=74.7%, *P*_异质性_=0.003); 3篇文献报道了两组与DBC的关联，结果显示同步治疗组DBC优于序贯治疗组(HR=0.77, 95%CI: 0.62-0.96, *I^2^*=80.5%, *P*_异质性_ < 0.001); 3篇文献报道了两组与RN/TRIC的关联，结果未发现两组间RN/TRIC具有统计学差异(*HR*=1.72, 95%CI: 0.85-3.47, *I^2^*=0%, *P*_异质性_=0.388)。详见图 3。

**图 3:**
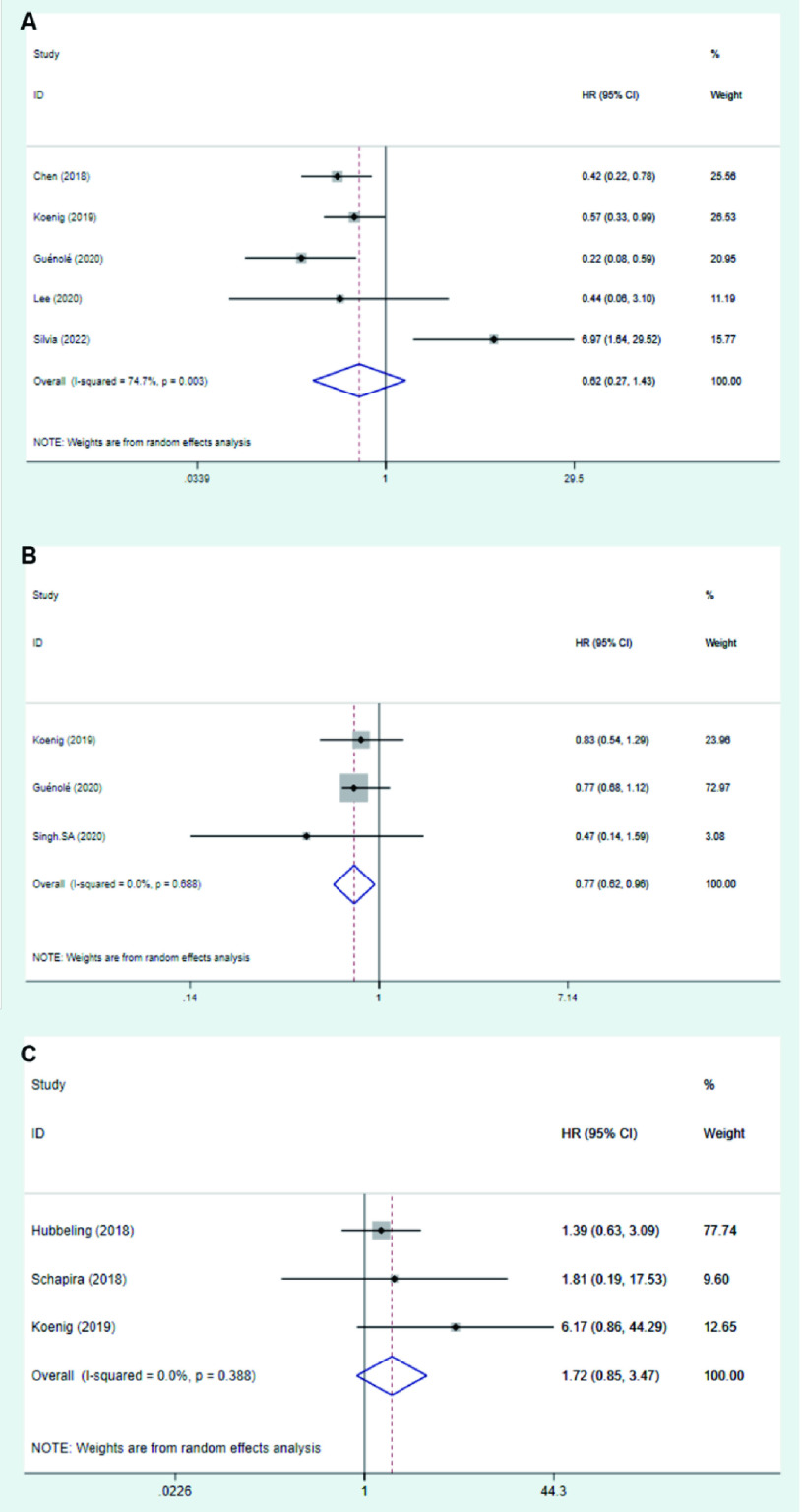
RT+IT同步治疗组 *vs* RT+IT序贯治疗组各结局的森林图。A：OS; B：DBC; C：RN/TIRC。 Forrest plot of synchronous treatment group *vs* RT+IT sequential treatment group. A: OS; B: DBC; C: RN/TIRC.

### 2.5 发表性偏倚及敏感性分析

研究数量 > 3篇时采用漏斗图评估纳入研究的发表偏倚。对于文献数≤3篇的结局不再进行发表偏倚和敏感性分析评价。RT+ICI和单独RT组比较中，OS、DBC和RN/TRIC结局的漏斗图基本对称，未发现明显的发表偏倚，其余组也未发现明显的发表偏倚。敏感性分析结果显示，删除任一篇文献对剩余文献合并效应值均无明显影响，证实了本研究最终结果的稳定性。见图 4。

**图 4:**
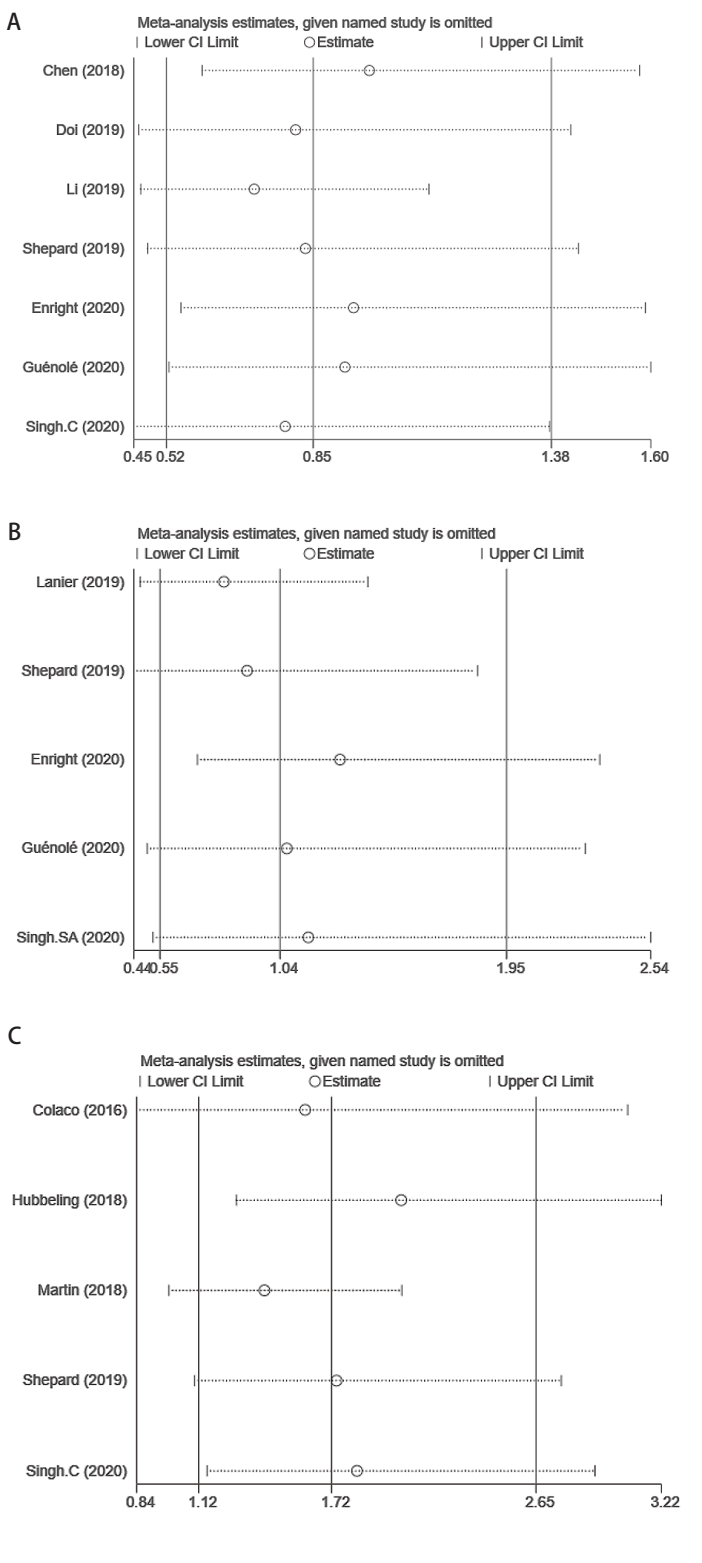
RT+IT组 *vs* RT组各结局的敏感图 A：OS; B：DBC; C：RN/TIRC。 Sensitivity plot of RT+IT *vs* RT group. A: OS; B: DBC; C: RN/TIRC.

## 3 讨论

本研究纳入17篇文献评价RT联合IT对NSCLC BM患者的疗效和安全性。就RT+IT组和单独RT组的比较而言，RT与IT联合使用并未显著改善患者的OS和DBC，但RT+IT组LC优于RT组，发生RN/TRIC的风险高于RT组，说明对于单独放疗组，联合治疗组局部进展控制得到改善，但是放射性坏死的副作用增加; 就RT+IT同步治疗组和RT+IT序贯治疗组比较而言，两组间OS与RN/TRIC无显著差异，但RT+IT同步治疗组DBC优于序贯治疗组。

同Shepard^[17]^的研究结果相似，本研究也并未发现RT联合IT可改善患者OS。但有之前一项*meta*分析^[25]^显示RT联合IT治疗可提高BM患者的生存率，考虑到该*meta*分析中纳入了会议摘要，本研究仅纳入已发表文献，并更新了相关文献，同时，生存率与随访时间相关，不同研究间随访时间差别较大，因此可能导致了观察结局的不同，这一结果仍需要大样本量及前瞻性的随机对照试验来证实。

之前有研究^[26, 27]^发现RT通过多种机制与IT产生协同作用，包括刺激肿瘤抗原的释放、增强抗原呈递细胞的活化、增加血脑屏障的通透性和上调IT靶向的细胞表面分子。两项主要涉及黑色素瘤患者的回顾性研究^[28, 29]^表明，RT与IT同步治疗可使相关的疾病结果有所改善。然而，鉴于正常组织也表达PD-L1以防止T细胞介导的正常组织损伤^[30]^，因此仍然存在同时进行RT和IT也可能导致症状性毒性风险增加的担忧。RT的主要严重副作用是RN/TRIC。一项回顾性研究^[31]^表明SRS与并发ICI可能会改善黑色素瘤患者的治疗结果，但可能会以增加发生症状性放射性坏死(radiation necrosis, RN)的风险为代价。Martin等^[11]^也发现接受RT和IT治疗的患者更容易出现RN，这一结果在另外两项*meta*分析^[25, 32]^中也得到证实。尽管RN的发病机制尚未完全确定，但可能与促炎机制的激活、血管损伤和血管生成异常有关。

综上所述，RT联合IT并未显著改善患者的OS，同时使患者暴露于较高的RN/TRIC发生风险中，因此建议慎重使用该联合治疗方案，对于是否对不同类型肺癌患者的治疗结局不同，仍需要后期RCT试验证实。目前有两项Ⅱ期试验(NCT04291092、NCT04787185)正在研究RT联合IT治疗的临床结果。还有一项Ⅱ期试验(NCT04650490)正在研究SRS后进行IT与SRS前进行IT治疗的结局差异。这些Ⅱ期试验的结果将为NSCLC BM患者的标准治疗提供启示。

本研究也存在局限性。首先，本研究结局均与随访时间相关，时间越长可能结局发生率相应增高，如RN可能在放疗后6个月-30个月出现，但纳入研究的中位随访时间存在差异，导致结果可能存在偏差。其次，纳入的文献均为回顾性队列研究，不同研究对照体系不同，导致结果有偏差。第三，虽然本研究中同步治疗组的时间间隔可以为1周-3个月，但联合RT加ICI组的时间间隔不能标准化，因为大多数原始研究没有定义这个时间间隔。最后，RT和IT分别有很多种类，但是由于相关研究较少，不能进一步分析不同种类对结局效应的影响。但是由于目前缺乏前瞻性的RCT研究支持RT联合IT在NSCLC脑转移患者中的作用，本研究分析可以为今后开展随机对照试验(randomized controlled trial, RCT)以及临床实践提供理论证据。
